# Relationship of Postoperative Temporary Facial Nerve Dysfunction With Tumor Location in Parotid Surgery

**DOI:** 10.7759/cureus.106344

**Published:** 2026-04-02

**Authors:** Shihab Asfar Khan, Kazi Shameemus Salam, Mohammad Habibur Rahman, Nayeemun Akter, Mohammad Hanif, Jonayed Sharker, Nazmus Sakib

**Affiliations:** 1 Otolaryngology and Head-Neck Surgery, National Institute of ENT, Dhaka, BGD; 2 Otolaryngology and Head-Neck Surgery, Bangladesh Medical University, Dhaka, BGD; 3 General Surgery, Bangladesh Medical University, Dhaka, BGD; 4 Otolaryngology and Head-Neck Surgery, Specialized ENT Hospital of SAHIC (SENTH), Dhaka, BGD

**Keywords:** parotidectomy, risk factors, surgical approach, temporary facial nerve dysfunction, tumor location

## Abstract

Introduction

Postoperative temporary facial nerve dysfunction (TFND) is a common complication after parotidectomy, and the tumor location is believed to influence its risk. This study aimed to evaluate the relationship between tumor location and postoperative TFND, as well as to assess the effects of lobe involvement and surgical approach.

Methods

This prospective observational study was conducted at Bangladesh Medical University, Dhaka, between June 2022 and December 2023, and 35 patients with parotid neoplasms who underwent surgery were enrolled in this study. Preoperative magnetic resonance imaging was used to determine the tumor location (anterior/posterior, superior/inferior, superficial/deep), and lobe involvement was confirmed intraoperatively. Facial nerve function was assessed via the House-Brackmann grading system on postoperative day 2 and at one and three months. Univariate and multivariate logistic regression were performed to analyze the TFND risk factors.

Results

The participants had a mean age of 48.80 ± 11.92 years, with a female predominance. Pleomorphic adenoma was the most common tumor type (60%). TFND occurred in 54.3% of patients on postoperative day 2, 51.4% at one month, and 45.7% at three months, most commonly involving the marginal mandibular branch. On multivariate analysis, anterior tumor location (OR: 25.05; 95% CI: 1.29-484.05; p = 0.033), bilobar involvement (OR: 21.84; 95% CI: 1.13-421.84; p = 0.041), and total conservative parotidectomy (OR: 20.89; 95% CI: 1.05-413.72; p = 0.046) were significantly associated with increased risk of TFND on postoperative day 2. At one month, anterior tumor location remained a significant predictor (OR: 31.75; 95% CI: 2.02-497.19; p = 0.014). At three months, anterior tumor location (OR: 13.59; 95% CI: 1.72-107.22; p = 0.013) and bilobar involvement (OR: 10.14; 95% CI: 1.39-73.92; p = 0.022) remained independently associated with persistent TFND. No significant association was observed between TFND and age, sex, tumor size, or histopathology.

Conclusion

Anterior tumor location, bilobar involvement, and extensive surgery significantly increase TFND risk and persistence. These findings suggest that careful preoperative imaging-based localization and meticulous surgical planning may help reduce the risk of TFND in parotid surgery.

## Introduction

Salivary gland neoplasms have become a major concern for head and neck surgeons. Tumors of the salivary glands account for 3-10% of neoplasms of the head and neck [1]. Most of these tumors affect the major salivary glands; however, they can occur anywhere [2]. The parotid gland is most frequently afflicted, with a range of 36.6% to 83%. The incidence of malignant tumors ranges from 15% to 32% [1,3]. Mucoepidermoid carcinoma and pleomorphic adenoma are the two most prevalent types of tumors, with the former being malignant and the latter being benign. Approximately 45-60% of all salivary gland tumors are pleomorphic adenomas. More than eighty percent of these cases are found in the parotid gland, most commonly in the superficial lobe; however, it can also occur in its deeper lobe or in the accessory parotid tissue less frequently [4].

Parotidectomy is the surgical removal of the parotid gland in either a partial or complete fashion. Inflammatory diseases, certain infectious processes, congenital anomalies, benign or malignant neoplasms, and other conditions might all be surgical candidates for various reasons [5]. Surgical procedures to remove tumors from the cheek or infratemporal fossa, in which it is desirable to keep the facial nerve and its branches intact, may include partial parotidectomy as a component of the procedure. Regardless of the reason for the surgery, parotidectomy is a difficult procedure that necessitates the use of expert surgeons because of the close interaction between the gland and the facial nerve [6]. Because of the close relationship between the parotid gland and the extrateporal path of the facial nerve, identifying the facial nerve trunk is critical during parotid gland surgery [7]. A proper incision, as well as the identification of multiple anatomical landmarks, is also needed to localize the facial nerve accurately to avoid facial dysfunction, which can be a potentially devastating condition that can have a significant impact on a patient's overall quality of life [7]. Additional typical clinical stages in parotidectomy include preoperative imaging tests, intraoperative electromyographic monitoring of the facial nerve, and the use of a magnified surgical view [7].

Magnetic resonance imaging (MRI) is a medical imaging technique used in radiology to form pictures of the anatomy and physiological processes of the body. MRI scanners use strong magnetic fields, magnetic field gradients, and radio waves to generate images of organs in the body. MRI does not involve X-rays or ionizing radiation, which distinguishes it from CT and PET [8]. MRI is widely used in hospitals and clinics for medical diagnosis, staging, and follow-up of diseases. Compared with CT, MRI provides better contrast in images of soft tissues, nerves, etc. Moreover, MRI (94.2%) provides better results in locating parotid tumors than CT (88.7%) in terms of the agreement between the preoperative prediction and the actual location of the tumor [9].

Facial weakness of one or more facial subsites for ≥6 months after parotidectomy can be defined as permanent facial palsy [7]. However, facial weakness of one or more facial subsites for less than five months after parotidectomy can be defined as temporary facial palsy/dysfunction [7]. According to earlier studies, 0 to 9% of patients experienced acute facial nerve dysfunction and persistent facial nerve impairment following parotidectomy [10]. Facial nerve injury, which affects mainly the marginal mandibular branch, is the most frequent neurologic complication from parotidectomy [11]. Siddiqui et al. reported that damage to the mandibular, buccal, and temporo-zygomatic branches was observed in 10 (14%), two (3%), and one (1.4%) patients [12], respectively, after parotidectomy. Ikoma et al. reported that the marginal mandibular nerve was the most affected branch of the facial nerve following parotidectomy [13].

The rate of facial nerve paresis is significantly greater in large-scale surgery (complete parotidectomy) and for tumors that are deeper than the facial nerve plane [13]. The results of univariate analysis revealed that age, malignant tumor, total parotidectomy, tumor size, and tumors located deeper than the facial nerve and crossing the nerve (i.e., located in both the superficial and deep lobes) were statistically significant risk factors for temporary facial weakness [14]. Moreover, tumor location, tumor margin, tumor size, and high tumor grade were also found to be significant factors related to facial nerve invasion (FNI) [14]. In addition, a study reported that the incidence of TFND in upper anterior and deep tumors was significantly greater than that in lower posterior and superficial tumors [13]. Therefore, the primary objective of this study was to evaluate the relationship between tumor location and postoperative temporary facial nerve dysfunction (TFND). The secondary objectives were to assess the effects of tumor lobe involvement and surgical approach on the occurrence and persistence of TFND.

## Materials and methods

Study design and setting

This prospective observational study was conducted in the Department of Otolaryngology - Head and Neck Surgery at Bangladesh Medical University, Dhaka, Bangladesh, between June 2022 and December 2023. Patients presenting with parotid gland neoplasms who were scheduled for parotid surgery during the study period were screened for eligibility. A total of 35 patients fulfilling the predefined inclusion and exclusion criteria were enrolled using a purposive sampling technique. Ethical approval for the study was obtained from the Institutional Review Board of Bangladesh Medical University, previously known as Bangabandhu Sheikh Mujib Medical University (approval no: BSMMU/2022/5053).

Sample size calculation

The minimum required sample size was calculated via the following formula:

\[ n = \frac{\left[ Z_{\alpha}\sqrt{P_{0}(100 - P_{0})} + Z_{\beta}\sqrt{P(100 - P)} \right]^2}{(P - P_{0})^2}, \]

assuming an anticipated proportion (P) of 19% and a standard proportion (P₀) of 39% based on previous literature [7], with a 95% confidence level (Zα = 1.96) and 80% power (Zβ = 0.85). This yielded a minimum sample size of 35 cases. Therefore, a total of 35 participants were included in the study. During the study period, no patients were lost to follow-up.

Inclusion and exclusion criteria

Patients with clinically suspected parotid gland neoplasms who were scheduled for surgical management at the study center during the study period were included in the study. Patients with preoperative facial nerve paralysis, a previous history of parotid surgery, known neurological disorders affecting facial nerve function, or those unwilling to undergo MRI evaluation were excluded from the study.

Preoperative evaluation and tumor localization

Baseline demographic characteristics, personal habits, and tumor-related variables (including location, size, nature, lobe involvement, and quadrant location) were recorded for each participant. Tumor location was determined preoperatively using MRI, selected for its superior soft tissue contrast and its ability to accurately delineate tumor extent and its relationship to the facial nerve and adjacent vascular structures. Although ultrasonography is commonly used as an initial imaging modality for salivary gland evaluation, it is operator-dependent and has limited ability to assess deep lobe involvement and complex anatomical relationships. Therefore, MRI was preferred in this study to provide more precise and reproducible anatomical localization for surgical planning. Tumors were classified as anterior or posterior and superior or inferior according to the anatomical reference lines described by Nishimura et al. Intraoperatively, tumors were further categorized as superficial, deep, or involving both lobes based on their relationship to the facial nerve [15]. Bilobar involvement was defined as tumors extending across both the superficial and deep lobes of the parotid gland, as confirmed intraoperatively based on their relationship to the facial nerve trunk. In addition, tumor location was interpreted in relation to the anatomical course of facial nerve branches; anterior and superior tumors were considered to be in closer proximity to the temporozygomatic and buccal branches, whereas inferior tumors were more closely related to the marginal mandibular branch.

Surgical procedure

All patients underwent parotidectomy under general anesthesia using standard surgical techniques. Facial nerve identification was carried out using established anatomical landmarks, including the tragal pointer, posterior belly of the digastric muscle, and tympanomastoid suture. Borle’s triangle, formed by the posterior belly of the digastric muscle, the mastoid process, and the angle of the mandible, was also used as an additional landmark to aid in locating the facial nerve trunk. Intraoperative facial nerve monitoring was performed using an electromyographic monitoring system, providing continuous visual and auditory feedback to assist in nerve identification and preservation. Depending on tumor characteristics and intraoperative findings, either superficial parotidectomy or total conservative parotidectomy was performed. The choice of procedure was based on tumor location, extent, and its relationship to the facial nerve. All procedures were performed by experienced surgeons with more than 10 years of surgical experience.

Outcome assessment

Postoperative facial nerve function was evaluated using the House-Brackmann grading system. Facial nerve dysfunction was categorized according to the House-Brackmann grading system, where Grade I represents normal function and Grades II-VI represent increasing severity of dysfunction. Assessments were performed on the second postoperative day and during follow-up visits at one month and three months after surgery. TFND was defined as postoperative facial nerve weakness expected to recover within six months. When dysfunction occurred, the specific branches involved were documented. Assessments were performed by the operating surgeons, and blinding was not feasible due to the nature of postoperative clinical evaluation. Postoperative complications and histopathological diagnoses were also recorded.

Data collection

Clinical and operative data were collected using a structured data collection form. Variables recorded included age, sex, personal habits, tumor location (anterior/posterior and superior/inferior), tumor size, tumor nature (benign or malignant), lobe involvement, quadrant location, type of surgery performed, histopathological diagnosis, and postoperative facial nerve function outcomes at each follow-up time point.

Statistical analysis

Data were analyzed using the Statistical Package for the Social Sciences (SPSS) (IBM SPSS Statistics for Windows, IBM Corp., Version 23, Armonk, NY). Continuous variables were summarized as mean ± standard deviation, whereas categorical variables were presented as frequency and percentage. Associations between categorical variables were assessed using the chi-square test or Fisher’s exact test, as appropriate. Univariate and multivariate logistic regression analyses were performed to identify potential risk factors associated with postoperative TFND at each follow-up time point. Odds ratios (ORs) with 95% confidence intervals (CIs) were calculated. A p-value of less than 0.05 was considered statistically significant.

**Figure 1:**
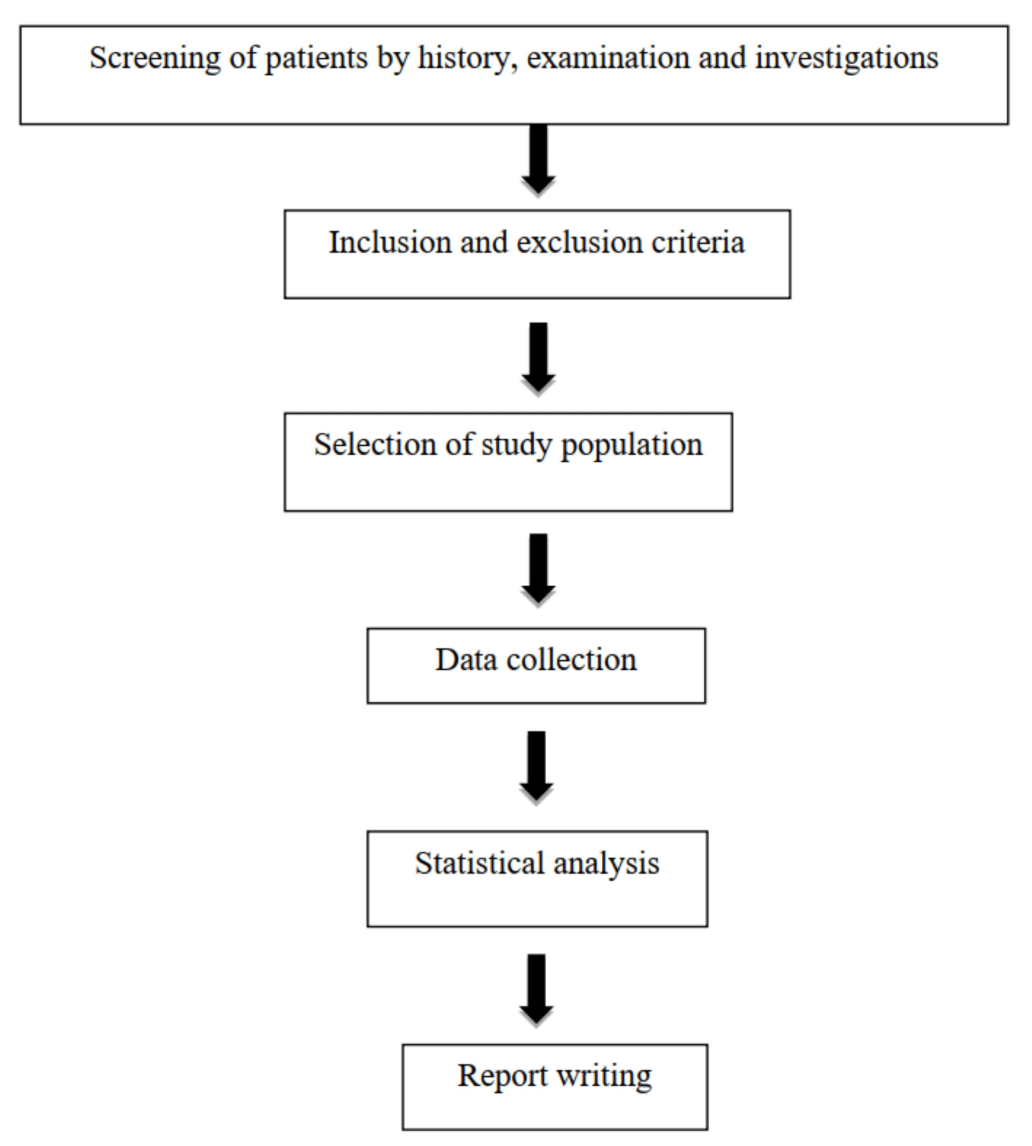
Study flowchart Image was created using Microsoft Word (Microsoft® Corp., Redmond, WA).

The study flowchart is presented in Figure 1.

## Results

The mean age of the patients was 48.8 ± 11.9 years, and a slight female predominance was noted (54.3%). Most patients were housewives (54.3%), with smaller proportions working as businessmen, farmers, teachers, or in private jobs. With respect to personal habits, 20.0% reported a history of smoking, and 25.7% reported chewing betel nut. The majority of patients presented with facial swelling (94.3%), followed by salivation problems (20.0%) and neck swelling (17.1%). Histopathological examination revealed that pleomorphic adenoma was the most common tumor type and was observed in 21 (60.0%) patients, followed by mucoepidermoid carcinoma in five (14.3%), Warthin tumors in four (11.4%), and other histologies in five (14.3%) (Table 1).

**Table 1:** Demographic and clinical characteristics of the study patients (n = 35)

Variable	Category	Frequency (%)
Age (years)	Mean ± SD	48.8 ± 11.9
Gender	Male	16 (45.7)
	Female	19 (54.3)
Occupation	Housewife	19 (54.3)
	Businessman	5 (14.3)
	Farmer	5 (14.3)
	Teacher	5 (14.3)
	Private job	1 (2.8)
Personal habits	Smoking	7 (20.0)
	Betel nut chewing	9 (25.7)
Presenting complaints	Facial swelling	33 (94.3)
	Salivation problems	7 (20.0)
	Neck swelling	6 (17.1)
Histological diagnosis	Pleomorphic adenoma	21 (60.0)
	Mucoepidermoid carcinoma	5 (14.3)
	Warthin tumor	4 (11.4)
	Others	5 (14.3)

Postoperative facial nerve dysfunction (TFND) was a frequent finding in the early postoperative period and gradually resolved over time (Table 2). On the second postoperative day, 19 patients (54.3%) underwent TFND, which declined slightly to 18 patients (51.4%) at one month and 16 patients (45.7%) at three months.

**Table 2:** Facial nerve dysfunction at different durations following parotid surgery (n = 35) TFND, temporary facial nerve dysfunction

Time Point	Normal (Grade I) n (%)	TFND Present n (%)
Second postoperative day	16 (45.7)	19 (54.3)
One month postoperative	17 (48.6)	18 (51.4)
Three months postoperative	19 (54.3)	16 (45.7)

Analysis of the specific nerve branches affected revealed that the marginal mandibular branch was the most commonly involved branch, being affected in isolation in 12 (63.2%) of the TFND patients on day 2, 16 (88.9%) at one month, and 15 (93.8%) at three months. In contrast, only a minority of patients demonstrated dysfunction involving multiple branches (Table 3).

**Table 3:** Branches of facial nerve injury following parotid surgery (n = 35)

Time Point	Marginal Mandibular Only n (%)	Multiple Branches n (%)
Second postoperative day	12 (63.2)	7 (36.8)
One month postoperative	16 (88.9)	2 (11.1)
Three months postoperative	15 (93.8)	1 (6.3)

The relationships between postoperative TFND and tumor characteristics, as well as the surgical approach, are summarized in Table 4. Among patients with anteriorly located tumors, 80% developed TFND on the second postoperative day, whereas only 20% of those with posterior tumors did. Similarly, TFND persisted in 70% of anterior tumor cases even at three months, whereas only 13.3% of posterior tumors presented dysfunction at that time. Tumors involving both superficial and deep lobes were associated with TFND in 83.3% of patients on the second postoperative day, whereas they were associated with TFND in 13.3% of patients with tumors confined to the superficial lobe. This pattern remained at three months, with 72.2% of bilobar tumors showing TFND compared with 13.3% of superficial lobe tumors. Superiorly located tumors had TFND rates of 78.6% on day 2 and 64.3% at three months, whereas inferior tumors had lower rates of 38.1% and 33.3%, respectively. In terms of surgery type, 92.3% of patients who underwent total conservative parotidectomy experienced TFND on the second postoperative day, whereas 31.8% of those who underwent superficial parotidectomy did; at three months, TFND persisted in 69.2% of total parotidectomy cases versus 31.8% of superficial cases. Tumor size and tumor nature (benign vs. malignant) showed less marked differences, although malignant tumors had higher rates of TFND at all time points.

**Table 4:** Association of TFND with tumor location and surgery (n = 35) TFND, temporary facial nerve dysfunction

Factor	Category	TFND Present on Second Postoperative Day n (%)	Test Statistic (χ²)	p-value	TFND Present at One Month n (%)	Test Statistic (χ²)	p-value	TFND Present at Three Months n (%)	Test Statistic (χ²)	p-value
Tumor site	Anterior (n = 20)	16 (80.0)	12.07	<0.001	15 (75.0)	12.80	<0.001	14 (70.0)	10.50	0.001
	Posterior (n = 15)	3 (20.0)	-	-	2 (13.3)	-	-	2 (13.3)	-	-
Tumor location	Superior (n = 14)	11 (78.6)	5.52	0.019	11 (78.6)	6.85	0.009	9 (64.3)	3.24	0.072
	Inferior (n = 21)	8 (38.1)	-	-	7 (33.3)	-	-	7 (33.3)	-	-
Tumor lobe involvement	Both superficial and deep (n = 18)	15 (83.3)	14.29	<0.001	14 (77.8)	13.02	<0.001	13 (72.2)	11.58	0.003
	Superficial (n = 15)	2 (13.3)	-	-	2 (13.3)	-	-	2 (13.3)	-	-
	Deep (n = 2)	2 (100.0)	-	-	2 (100.0)	-	-	1 (50.0)	-	-
Tumor volume	Small (n = 18)	7 (38.9)	3.53	0.060	7 (38.9)	2.33	0.127	6 (33.3)	2.29	0.130
	Large (n = 17)	12 (70.6)	-	-	11 (64.7)	-	-	10 (58.8)	-	-
Nature of tumor	Benign (n = 28)	13 (46.4)	3.48	0.062	12 (42.9)	4.13	0.042	11 (39.3)	2.33	0.127
	Malignant (n = 7)	6 (85.7)	-	-	6 (85.7)	-	-	5 (71.4)	-	-
Type of operation	Superficial parotidectomy (n = 22)	7 (31.8)	10.67	0.001	7 (31.8)	8.90	0.003	7 (31.8)	4.58	0.032
	Total conservative parotidectomy (n = 13)	12 (92.3)	-	-	11 (84.6)	-	-	9 (69.2)	-	-

The multivariate analysis in Table 5 confirmed these patterns. On the second postoperative day, anterior tumor location increased the odds of TFND by 25 times (OR: 25.05), bilobar tumor involvement by 21.84 times (OR: 21.84), and total conservative parotidectomy by 20.89 times (OR: 20.89) compared with the respective reference groups. At one month, anterior tumor location was associated with a 31.75-fold greater odds of TFND (OR: 31.75). By three months, anterior tumor location and bilobar involvement remained significant predictors, with ORs of 13.59 and 10.14, respectively.

**Table 5:** Univariate and multivariate logistic regression analysis of risk factors for TFND (n = 35) TFND, temporary facial nerve dysfunction

Time point	Risk factor	Unadjusted OR	95% CI	p-value	Adjusted OR	95% CI	p-value
Second postoperative day	Tumor lobe (deep and superficial)	16.25	3.05-86.41	0.001	21.84	1.13-421.84	0.041
	Tumor site (anterior)	16.00	3.00-85.30	0.001	25.05	1.29-484.05	0.033
	Tumor site (superior)	5.95	1.26-28.00	0.024	4.37	0.25-75.73	0.310
	Total conservative parotidectomy	25.74	2.76-238.79	0.004	20.89	1.05-413.72	0.046
One month postoperative	Tumor lobe (deep and superficial)	11.35	2.34-55.12	0.003	12.67	0.81-197.09	0.070
	Nature of tumor (malignant)	8.00	0.84-75.55	0.070	-	-	-
	Tumor site (anterior)	26.00	4.09-165.09	0.001	31.75	2.02-497.19	0.014
	Tumor site (superior)	7.33	1.53-35.11	0.013	6.65	0.41-106.09	0.180
	Total conservative parotidectomy	11.78	2.04-68.06	0.006	7.95	0.59-106.00	0.117
Three months postoperative	Tumor lobe (deep and superficial)	12.13	2.40-61.20	0.003	10.14	1.39-73.92	0.022
	Tumor site (anterior)	15.16	2.58-88.99	0.003	13.59	1.72-107.22	0.013
	Total conservative parotidectomy	4.82	1.09-21.19	0.037	1.76	0.25-12.37	0.569

## Discussion

This study, conducted at a tertiary care hospital, provides an in-depth assessment of the prevalence and risk factors for TFND following parotid surgery, highlighting the significant impact of tumor location and surgical approach on postoperative outcomes.

Our study revealed that pleomorphic adenoma was the most common type, followed by mucoepidermoid carcinoma and Warthin tumors. Tumors located in deep or upper/anterior regions are associated with increased rates of TFND. This finding aligns with prior studies demonstrating that tumor location relative to facial nerve branches significantly influences postoperative neuropraxia [16,17]. For example, Ikoma et al. reported more transient nerve weakness in tumors extending into the deep lobe [13], whereas another study noted similar patterns [18]. Other studies also emphasized tumor location over histology as a key risk factor [7,19,20]. This may be due to increased nerve manipulation during deep-lobe dissections and anatomical proximity to the nerve trunk. Therefore, precise preoperative imaging and intraoperative nerve monitoring are essential to minimize this risk.

The findings of this study also indicate that postoperative facial nerve dysfunction is common immediately after surgery, with gradual improvement over time. The marginal mandibular branch emerged as the most frequently affected branch, with most cases showing isolated involvement and recovery by three months. This pattern echoes the findings of numerous studies reporting that transient paresis commonly affects the marginal mandibular branch, often with full resolution within months [21]. Some studies also note the occasional involvement of additional branches. The predominance of marginal mandibular involvement likely reflects its superficial anatomical course and vulnerability to traction or compression during dissection [22,23]. The microsurgical technique and intraoperative nerve monitoring, as supported by prior work, may reduce injury and facilitate recovery [24]. Thus, careful handling of this branch and enhanced visualization techniques are recommended to minimize transient dysfunction.

Our findings revealed that tumors located anteriorly, superiorly, bilobarly, or requiring total conservative parotidectomy were strongly associated with prolonged TFND, whereas posterior, inferior, and superficial lobe tumors presented milder, more transient dysfunction. This underscores the influence of anatomical proximity and surgical complexity on nerve outcomes. These observations mirror several studies: Kojima et al. noted greater TFND in anterior, superior, and deep lobe tumors [24]; Jeong et al. reported similar associations [25]; and European multicenter research confirmed the impact of deep location [26]. Conversely, some reports have suggested that tumor size and malignancy are relatively few factors [13,27]. The probable mechanism is increased nerve manipulation during the extensive or complex dissections required for challenging tumor locations. Preoperative mapping and meticulous dissection with nerve monitoring are thus vital for reducing TFND risk.

Multivariate analysis revealed that anterior tumor location, bilobar involvement, and extensive surgery markedly increase both the risk and persistence of TFND, with anterior tumor location appearing to be consistently associated with a higher risk of TFND over time. These findings underscore that anatomical proximity and surgical complexity are major determinants of nerve outcomes. These findings align with earlier studies: Yokohama City University reported increased odds of TFND for anterior, upper, and deep-lobe tumors [13,26]; a 2019 BMC surgery review identified deep and bilobar tumors as independent predictors; and Kwon et al. confirmed deep tumor location and malignancy as risk factors [27]. Conversely, some machine-learning analyses have emphasized patient factors over tumor location [28]. The probable reasons may include increased nerve traction, manipulation, and exposure during the resection of anterior, bilobar, or large tumors. Enhanced preoperative imaging and intraoperative nerve monitoring are therefore critical to mitigate these risks.

This study has several limitations. It was conducted at a single tertiary care center with a relatively small sample size, which may limit generalizability and reduce the stability of the multivariate analysis. The use of purposive sampling may introduce selection bias. Postoperative facial nerve function was assessed by the operating surgeons without blinding, and inter-rater reliability of the House-Brackmann grading system was not evaluated, which may introduce observer bias. Although experienced surgeons performed all procedures, some variability in surgical technique cannot be excluded. Furthermore, tumor location classification, although based on established anatomical references and intraoperative findings, may be subject to some variability. Not all relevant tumor- and surgery-related variables were fully accounted for, which may contribute to residual confounding. The follow-up period was limited to three months, restricting long-term outcome assessment. Therefore, the findings should be interpreted with caution, and larger multicenter studies with longer follow-up are needed to validate these results. Future multicenter studies with larger sample sizes and longer follow-up periods are recommended to validate these findings. Advanced imaging techniques and intraoperative nerve monitoring strategies should also be further explored to reduce the incidence of TFND.

## Conclusions

Tumor location and surgical approach appear to influence the occurrence and persistence of postoperative TFND in patients undergoing parotidectomy. Patients with anteriorly located tumors, bilobar involvement, and those undergoing total conservative parotidectomy tended to have higher rates of TFND in both the early postoperative period and at three months. The marginal mandibular branch was the most frequently affected branch, with gradual recovery observed in most cases. These findings suggest that careful preoperative imaging-based localization and meticulous intraoperative dissection may help reduce the risk of nerve injury. However, given the study limitations, these results should be interpreted with caution. Further multicenter studies with larger sample sizes and longer follow-up are needed to validate these findings and to explore strategies for improving nerve recovery in high-risk patients.

## References

[1] Foresta E, Torroni A, Di Nardo F (2014). Pleomorphic adenoma and benign parotid tumors: extracapsular dissection vs superficial parotidectomy—review of literature and meta-analysis. Oral Surg Oral Med Oral Pathol Oral Radiol.

[2] Singh Nanda KD, Mehta A, Nanda J (2012). Fine-needle aspiration cytology: a reliable tool in the diagnosis of salivary gland lesions. J Oral Pathol Med.

[3] Satko I, Stanko P, Longauerová I (2000). Salivary gland tumours treated in the stomatological clinics in Bratislava. J Craniomaxillofac Surg.

[4] Zbären P, Vander Poorten V, Witt RL (2013). Pleomorphic adenoma of the parotid: formal parotidectomy or limited surgery?. Am J Surg.

[5] Wang SJ, Eisele DW (2012). Parotidectomy—anatomical considerations. Clin Anat.

[6] O'Brien CJ (2003). Current management of benign parotid tumors—the role of limited superficial parotidectomy. Head Neck.

[7] Jin H, Kim BY, Kim H (2019). Incidence of postoperative facial weakness in parotid tumor surgery: a tumor subsite analysis of 794 parotidectomies. BMC Surg.

[8] (2026). Magnetic Resonance Imaging (MRI). https://www.nibib.nih.gov/science-education/science-topics/magnetic-resonance-imaging-mri.

[9] Vaiman M, Luckman J, Sigal T, Bekerman I (2016). Correlation between preoperative predictions and surgical findings in the parotid surgery for tumors. Head Face Med.

[10] Saha S, Pal S, Sengupta M, Chowdhury K, Saha VP, Mondal L (2014). Identification of facial nerve during parotidectomy: a combined anatomical & surgical study. Indian J Otolaryngol Head Neck Surg.

[11] Costa MG e ST da, Maranhão-Filho P de A, Santos IC, Luiz RR, Vincent MB (2019). Parotidectomy-related facial nerve lesions: proposal for a modified Sunnybrook Facial Grading System. Arq Neuro-Psiquiatr.

[12] Siddiqui AH, Shakil S, Rahim DU, Shaikh IA (2020). Post parotidectomy facial nerve palsy: a retrospective analysis. Pak J Med Sci.

[13] Ikoma R, Ishitoya J, Sakuma Y, Hirama M, Shiono O, Komatsu M, Oridate N (2014). Temporary facial nerve dysfunction after parotidectomy correlates with tumor location. Auris Nasus Larynx.

[14] Cho WK, Lee MK, Choi YJ, Lee YS, Choi SH, Nam SY, Kim SY (2022). Preoperative magnetic resonance imaging and computerized tomography findings predictive of facial nerve invasion in patients with parotid cancer without preoperative facial weakness—a retrospective observational study. Cancers (Basel).

[15] Nishimura H, Kawata R, Kinoshita I, Higashino M, Terada T, Haginomori SI, Tochizawa T (2023). Proposal for a novel classification of benign parotid tumors based on localization. Auris Nasus Larynx.

[16] Suzuki S, Bandoh N, Goto T (2022). A retrospective study of parotid gland tumors at a single institution. Oncol Lett.

[17] Gautam SK, Kumar S, Singh HP, Singh AB, Chandra M (2023). Clinico-pathological profile of parotid gland tumors at a tertiary care center in North India. Natl J Maxillofac Surg.

[18] Savvas E, Hillmann S, Weiss D, Koopmann M, Rudack C, Alberty J (2016). Association between facial nerve monitoring with postoperative facial paralysis in parotidectomy. JAMA Otolaryngol Head Neck Surg.

[19] Cirignaco G, Monarchi G, Betti E (2025). Outcome of facial nerve integrity after parotid gland surgery with and without intraoperative monitoring: a ten-year retrospective study. J Clin Med.

[20] Gaillard C, Périé S, Susini B, St Guily JL (2005). Facial nerve dysfunction after parotidectomy: the role of local factors. Laryngoscope.

[21] Stodulski D, Skorek A, Mikaszewski B, Wiśniewski P, Stankiewicz C (2015). Facial nerve grading after parotidectomy. Eur Arch Otorhinolaryngol.

[22] Nakamura Y, Teramoto Y, Asami Y (2017). The rate of facial nerve dysfunction and time to recovery after intraparotid and extraparotid facial nerve exposure and protection in head and neck cutaneous tumor surgery. Int J Clin Oncol.

[23] Dasappa P, Bhagavan BC, Prajwal RK (2019). Does immediate post-operative severity of facial nerve palsy predict its recovery in parotidectomy patients - our experience. Int J Med Sci Curr Res.

[24] Kojima T, Matsumoto F, Suzuki Y, Sakyo A, Kojima M, Fujimaki M, Ohba S (2022). Comparison of different methods for evaluating the relationship between facial nerve and benign parotid tumors. SAGE Open Med.

[25] Jeong HS, Kim Y, Kim HJ (2023). Imaging of facial nerve with 3D-DESS-WE-MRI before parotidectomy: impact on surgical outcomes. Korean J Radiol.

[26] Moussa HR, Mlees MA, Abdelhamid AF (2021). Incidence and risk factors of facial nerve palsy after parotidectomy for benign parotid diseases. Egypt J Surg.

[27] Kwon J, Eom KY, Kim YS (2018). The prognostic impact of the number of metastatic lymph nodes and a new prognostic scoring system for recurrence in early-stage cervical cancer with high risk factors: a multicenter cohort study (KROG 15-04). Cancer Res Treat.

[28] Mundada AB, Pradeep P (2018). Comparison of antegrade versus retrograde facial nerve dissection in cases of superficial parotidectomy for pleomorphic adenoma of parotid gland. Int Surg J.

